# The Minimal Subcortical Electronic Threshold Predicts the Motor Deficit and Survivals in Non-Awake Surgery for Gliomas Involving the Motor Pathway

**DOI:** 10.3389/fonc.2022.789705

**Published:** 2022-03-15

**Authors:** Xiaohui Ren, Xiaocui Yang, Wei Huang, Kaiyuan Yang, Li Liu, Yong Cui, Lanjun Guo, Hui Qiao, Song Lin

**Affiliations:** ^1^ Neurosurgery, Beijing Tiantan Hospital, Capital Medical University, Beijing, China; ^2^ Beijing Neurosurgical Institute, Beijing, China; ^3^ National Clinical Research Center for Neurological Diseases, Beijing, China; ^4^ Institute for Brain Disorders and Beijing Key Laboratory of Brain Tumor, Beijing, China; ^5^ Surgical Neuromonitoring Service, University of California, San Francisco, CA, United States

**Keywords:** gliomas, motor pathway, high-frequency, subcortical stimulation, threshold, motor deficit, survivals

## Abstract

**Purpose:**

Direct subcortical motor mapping is the golden criterion to detect and monitor the motor pathway during glioma surgery. Minimal subcortical monopolar threshold (MSCMT) means the minimal distance away from the motor pathway and is critical to decide to continue or interrupt glioma resection. However, the optimal cutoff value of MSCMT for glioma resection in non-awake patients has not been reported discreetly. In this study, we try to establish the safe cutoff value of MSCMT for glioma resection and analyzed its relationship with postoperative motor deficit and long-term survivals.

**Methods:**

We designed this prospective study with high-frequency electronic stimulus method. The cutoff MSCMT of postoperative motor deficits was statistically calculated by receiver operating characteristic (ROC) curve, and its relationship with motor deficit and survivals was analyzed by logistic and Cox regression, respectively.

**Results:**

The cutoff MSCMT to predict motor deficit after surgery was 3.9 mA on day 1, 3.7 mA on day 7, 5.2 mA at 3 months, and 5.2 mA at 6 months. MSCMT ≤3.9 mA and MSCMT ≤5.2 mA independently predicted postoperative motor deficits at four times after surgery (*P* < 0.05) but had no effect on the removal degree of tumor (*P* > 0.05). In high-grade gliomas, MSCMT ≤3.9 mA independently predicted shorter progression-free survival [odds ratio (OR) = 3.381 (1.416–8.076), *P* = 0.006] and overall survival [OR = 3.651 (1.336–9.977), *P* = 0.012]. Power model has the best fitness for paired monopolar and bipolar high-frequency thresholds.

**Conclusions:**

This study showed strong cause–effect relation between MSCMT and postoperative motor deficit and prognoses. The cutoff MSCMT was dug out to avoid postoperative motor deficit. Further studies are needed to establish the results above.

## Highlights

1. The minimal subcortical monopolar threshold predicts postoperative motor deficit and survivals in glioma surgery.
2. Here, 3.9 mA is the safe cutoff MSCMT to predict motor deficit during operation procedure.
3. The power model is best fit for paired monopolar and bipolar thresholds.

## Importance of the study

The threshold monitored by direct subcortical stimulus means the minimal distance away from the motor pathway and is critical to decide to continue or interrupt glioma resection. However, the minimal threshold to interrupt resection has not been reported yet. In this study, we established the cutoff minimal threshold to avoid over-resection and analyzed its association with postoperative motor deficit and long-term survivals. We found that the cutoff minimal subcortical monopolar threshold (MSCMT) to predict motor deficit at different time points ranged from 3.7 to 5.2 mA. Lower MSCMT independently predicted postoperative motor deficits and shorter survivals.

## Introduction

Using electrophysiological techniques to monitor the motor pathway has gradually become the golden criterion for functional localization (1) and facilitated the maximal safe resection of gliomas. The traditional technique of bipolar stimulation at a low frequency, 50 or 60 Hz, has been used for decades and has been reported as an accurate and reliable technique for detecting the motor pathway (2). A new motor mapping method with high-frequency stimulation has also been reported and is increasingly being used widely because of the low probability of seizure (3–8). It has been stressed that high-frequency monopolar stimulation should be theoretically appropriate for monitoring the motor pathway located in deep cerebral tissue (9).

As the great importance of accurate neuro-location, the subcortical electronic stimulation threshold is usually used as a pivotal reference to decide whether the glioma resection should be continued or not. That value of minimal subcortical monopolar threshold (MSCMT) means the minimal distance away from the motor pathway. However, the quantitative relationship between threshold and distance is still equivocal (1–3, 5), and the cutoff minimal threshold for the brain–glioma’s vague interface is undefined either. To explore the optimal range of tumor resection guided by subcortical stimulus in non-awakened patients, we designed this study with high-frequency stimulus. Prospectively, we recorded monopolar subcortical thresholds during surgery, removal degree, motor deficit, and survivals after surgery. The cutoff minimal threshold was calculated by receiver operating characteristic (ROC) curve analysis, and logistic and Cox regression were also respectively used to demonstrate the relationship of MSCMT with postoperative motor deficit and long-term survivals.

## Materials and Methods

### Patient Characteristics

Seventy-nine patients with gliomas involving the motor pathway were enrolled in the study at Beijing Tiantan Hospital from 2015 to 2017. All patients provided written informed consent for the current study, and the clinical study was approved by the Medical Ethics Committee of Beijing Tiantan Hospital, Capital Medical University.

### Clinical and Radiological Records

We prospectively collected the following data: age, gender, tumor size and location, removal degree, pathology, postoperative motor function of the limbs, progression-free survival (PFS), and overall survival (OS). Tumor size defined as the maximal diameter was measured according to MR images. Tumor volume was computed from volumetric fluid-attenuated inversion recovery (FLAIR) MRI scans for low-grade gliomas and from post-contrast T1-weighted MRI scans for high-grade gliomas. The extent of resection (EOR) was determined according to the following equation: (preoperative tumor volume - postoperative tumor volume)/preoperative tumor volume (10, 11). An EOR ≥98% was defined as gross total resection (GTR). The PFS was defined as the duration between surgery and initial radiological recurrence. The OS was defined as the duration between surgery and death.

### Clinical Assessment of Motor Function

Motor function was assessed by a scale of 0–5 scores (0 = no contraction, 1 = flicker or trace contraction, 2 = movement with gravity eliminated, 3 = movement against gravity, 4 = movement against resistance, 5 = normal strength) at five different time points: 1) in the preoperative hospitalization period, 2) in the recovery room immediately after surgery, 3) at discharge from the hospital (7 days postoperatively), 4) 3 months after surgery, and 5) 6 months after surgery.

### Neurophysiology

Somatosensory evoked potentials (SEPs) and motor evoked potentials (MEPs) were recorded during the surgical procedures. A Nicolet Endeavor CR (Natus, USA) was used for intraoperative electrophysiological recording. Before craniotomy, bilateral tibial and median nerve SEPs were elicited by conventional stimulation at the ankle and wrist (25 mA; duration, 0.2 ms; 2.1 Hz) and recorded with scalp corkscrew electrodes placed over the primary somatosensory cortex. Transcranial MEPs were elicited with a constant-voltage stimulator (SP-1, Nicolet) using corkscrew electrodes placed over the primary motor cortex. Stimulation was performed using 8 pulses of 50–400 V, 500-ms pulse duration, and an interstimulus interval of 2 ms. Contralateral muscle responses were recorded with needle electrodes inserted in the orbicularis oris, trapezius, deltoids, wrist flexors, hypothenar and thenar muscles, abductor pollicis brevis, quadriceps, and tibialis anterior in a standardized fashion. Amplitudes and latencies for MEPs and SEPs of the recorded body parts were noted at the beginning and end of surgery. Measurements of SEPs from the median nerve were based on the peak of the N20 component, while measurements of SEPs from the tibial nerve were based on the trough of the P40 component. Intraoperative identification of the central sulcus and Brodmann area 4 was accomplished with a combination of SEP phase reversal and direct high-frequency electric stimulation on the cortex (8-pulse sequence train; duration, 500 µs; interstimulus interval, 2 ms; 500 Hz), with the frontal needle electrode placed at the FZ as the cathode. For direct stimulation, stimulating intensity was increased stepwise to a maximum of 40 mA or the level at which the MEPs were detected. The stimulating current threshold (mA) was determined by the lowest intensity resulting in a reproducible muscle response surpassing 50 µV in peak-to-peak amplitude. Anodal (positive-current) and cathodal (negative-current) stimuli were used for cortical and subcortical mapping, respectively. Bipolar stimulation following monopolar stimulation was performed at the same point, and the electrical current threshold was recorded in milliamperes (mA).

### Surgery and Anesthesia

Surgery was performed according to well-established standard techniques. General anesthesia was maintained intravenously with propofol and remifentanil. To avoid interference with neurophysiological monitoring, inhaled anesthetic agents and muscle-relaxing drugs were avoided after induction. In a few cases, very low doses of inhaled anesthetic agents were used.

Functional MR (fMR) was performed before surgery in most cases to reveal the primary motor cortex and eloquent areas, as well as 3-dimensional tractography based on diffusion tensor imaging (DTI) to reveal the descending motor pathway. Imaging data from fMR were analyzed offline and loaded into the neuro-navigation system (Brainlab, Feldkirchen, Germany) for preoperative planning and intraoperative navigation. For motor mapping, a strip electrode with 6 contacts (each 4 mm in diameter and with an interelectrode distance of 6 mm) was used for cortical and subcortical stimulation (Beijing HKHS Healthcare Co., Ltd., PSE-6). One contact was used as an active stimulus for intermittent monopolar stimulation. Intraoperative identification of the central sulcus and Brodmann area 4 was accomplished with a combination of SEP phase reversal and direct high-frequency electric stimulation on the cortex (8-pulse sequence train; duration, 500 µs; interstimulus interval, 2 ms; 500 Hz), with the frontal needle electrode placed at the FZ as the cathode. Anodal (positive-current) and cathodal (negative-current) stimuli were used for cortical and subcortical mapping, respectively. After the dura was opened, SEP phase reversal and direct cortical stimulation enabled localization of the primary motor areas. The tumor was real-time monitored with the help of intraoperative navigation and ultrasound and carefully dissected under continuous electrophysiological monitoring. Throughout the surgical resection, paired monopolar and bipolar thresholds at the same point were recorded during direct subcortical stimulation along the brain–tumor interface, which provided valuable information about cautious areas.

### Statistical Analysis

The ROC curve was used to calculate the cutoff threshold for diagnosis of postoperative motor deficit. The logistic regression model was used to analyze the relationship of independent factor with motor deficit. Log-rank method and Cox regression model were used in survival analysis. The statistical software SPSS 13.0 (SPSS for Windows, version 13.0; SPSS Inc., Chicago, IL, USA) was used. Probability values were obtained from 2-tailed tests, with statistical significance defined as *P* < 0.05.

## Results

### Clinical Features of 79 Patients With Gliomas Involving the Motor Pathway

Clinical data are summarized in **Supplementary Table S1**. The age of onset ranged from 14 to 75 years old; the mean age was 42 ± 14 years. The patients included 44 men (55.7%) and 35 women (44.3%). Preoperative Karnofsky performance scale (KPS) ranged from 40 to 100, with a median of 80. Twenty-six tumors were located in the insular lobe; 46 in the perirolantic areas in the frontal, temporal, or parietal lobe; and 7 in the thalamus. Seventy-one were primary gliomas, and 8 were recurrent. Pathological findings revealed 3 Grade I, 34 Grade II, 20 Grade III, and 22 Grade IV tumors. Tumor was totally removed in 47 cases, subtotally removed in 28 cases, and partially removed in 4 cases.

Intraoperative seizure, which was defined as persistent contralateral electromyogram signal following cessation of cortical stimulation, was found in only one case (1.3%). No other side effect was found during the process of electronic monitoring in the operation.

Minimal subcortical threshold in each case was recorded, which ranged from 0.1 to 27.0 mA (5.0 mA as the median).

Patients were followed for 8–52 months for the recovery of motor deficit and survivals. Eleven patients harbored motor deficit before surgery. Twenty-four patients suffered from new motor deficit on day 1, 4 of whom had a quick recovery within 7 days, 8 recovered by 3 months, and 2 recovered by 6 months after surgery.

### The Cutoff Minimal Subcortical Electronic Threshold to Evaluate Postoperative Motor Deficit

**Figure 1:**
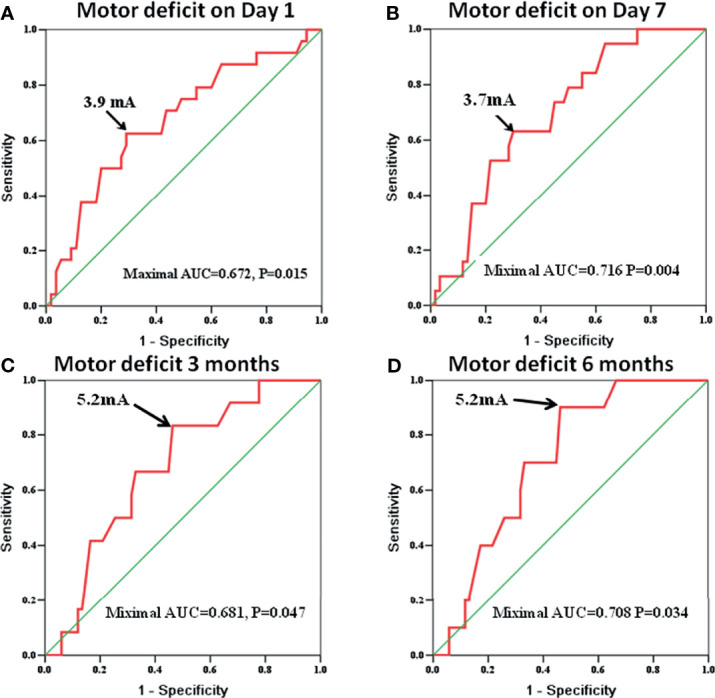
The receiver operating characteristic (ROC) curve identifies that the cutoff subcortical monopolar threshold to predict motor deficit was 3.9 mA on day 1 [**(A)**, *P* = 0.015], 3.7 mA on day 7 [**(B)**, *P* = 0.004], 5.2 mA at 3 months [**(C)**, *P* = 0.047], and 5.2 mA at 6 months after surgery [**(D)**, *P* = 0.034].

The MSCMTs and postoperative motor deterioration (yes/no at different time points after surgery) in each case were recorded. By ROC analysis, the cutoff MSCMT to predict postoperative motor deficit was shown at four different time points after surgery (N = 79, see **Supplementary Table S2**). The cutoff MSCMT 3.9 mA to predict motor deficit on day 1 after surgery (**Figure 1A**) showed that its sensitivity, specificity, positive predictive value (PPV), and negative predictive value (NPV) were 62.5%, 70.9%, 48.4%, and 81.3%, respectively. The maximal area under the curve (AUC) was 0.672 (95% CI, 0.541–0.804, *P* = 0.015). Meanwhile, the cutoff MSCMT 3.7 mA to predict motor deficit on day 7 after surgery (**Figure 1B**) displayed that the maximal AUC was 0.716 (95% CI, 0.595–0.837, *P* = 0.004). The cutoff MSCMT to predict motor deficit 3 months and 6 months after surgery was both 5.2 mA (**Figures 1C, D** **)**. The maximal AUC was 0.681 (95% CI, 0.536–0.826, *P* = 0.047) and 0.708 (0.573–0.843, *P* = 0.034), respectively. The detailed sensitivity, specificity, PPV, and NPV for different cutoff thresholds were shown in **Supplementary Table S2**.

### Independent Relation of Minimal Subcortical Monopolar Threshold With Postoperative Motor Deficit

The association between clinical factors and postoperative motor deficit was analyzed (**Tables 1**, **3**). Univariate analysis revealed that preoperative limb weakness, decreased transcranial MEPs, MSCMT ≤3.9 mA, and MSCMT ≤5.2 mA were correlated with postoperative motor deficit. Multivariable logistic regression model further confirmed that MSCMT ≤3.9 mA was reversely related with postoperative motor deficit on day 1 [3.478 (1.192–10.145), *P* = 0.022), on day 7 [4.230 (1.447–12.370), *P* = 0.008], 3 months [3.826 (1.041–14.065), *P* = 0.043], and 6 months [4.375 (1.036–18.473), *P* = 0.045]. Multivariable logistic regression model also further discovered that MSCMT ≤5.2 mA was reversely correlated with postoperative motor deficit on day 1 [3.125 (1.034–9.445), *P* = 0.043], on day 7 [3.688 (1.145–11.880), *P* = 0.029], 3 months [5.806 (1.181–28.540), *P* = 0.030], and 6 months [10.406 (1.250–86.650), *P* = 0.030].

**Table 1:** Factors correlated with postoperative motor deficit at different time points (n = 79).

Factors		Motor deficit after surgery							
		Day 1	*P* value	Day 7	*P* value	3 months	*P* value	6 months	*P* value
Age	<40 years	30.0% (12/40)	0.941	27.5% (11/40)	0.651	17.5% (7/40)	0.562	15.0% (6/40)	0.768
	≥40 years	30.8% (12/39)		23.1% (9/39)		12.8% (5/39)		10.3% (4/39)	
Gender	Men	29.5% (13/44)	0.857	22.7% (10/44)	0.553	13.6% (6/44)	0.666	11.4% (5/44)	0.962
	Women	31.4% (11/35)		28.6% (10/35)		17.1% (6/35)		14.3% (5/35)	
Primary/recurrent	Primary	29.6% (21/71)	0.955	23.9% (17/71)	0.684	14.1% (10/71)	0.767	11.3% (8/71)	0.585
	Recurrent	37.5% (3/8)		37.5% (3/8)		25.0% (2/8)		25.0% (2/8)	
Removal degree	GTR	23.4% (11/47)	0.102	21.3% (10/47)	0.317	12.8% (6/47)	0.683	10.6% (5/47)	0.757
	Non-GTR	40.6% (13/32)		31.3% (10/32)		18.8% (6/32)		15.6% (5/32)	
Pre-op. strength	Weaken	72.7% (8/11)	0.003*	54.5% (6/11)	0.042*	27.3% (3/11)	0.453	18.2% (2/11)	0.916
	Normal	23.5% (16/68)		20.6% (14/68)		13.2% (9/68)		11.8% (8/68)	
MSCMT**	≤3.90 mA	48.4% (15/31)	0.005*	41.9% (13/31)	0.006*	25.8% (8/31)	0.073	22.6% (7/31)	0.074
	>3.90 mA	18.8% (9/48)		14.6% (7/48)		8.3% (4/48)		6.3% (3/48)	
MSCMT**	≤5.20mA	41.5% (17/41)	0.026*	36.6% (15/41)	0.017*	24.4% (10/41)	0.018*	22.0% (9/41)	0.025*
	>5.20 mA	18.4% (7/38)		13.2% (5/38)		5.3% (2/38)		2.6% (1/38)	
Transcranial MEP	Unchanged	29.8% (14/47)	0.102#	23.4% (11/47)	0.066^#^	10.6% (5/47)	0.018*^#^	8.5% (4/47)	0.196^#^
	Decreased	100.0% (2/2)		100.0% (2/2)		100.0% (2/2)		50.0% (1/2)	
Transcranial SEP	Unchanged	30.2% (13/43)	1.000	23.3% (10/43)	0.684	11.6% (5/43)	0.653	9.3% (4/43)	1.000^#^
	Decreased	37.5% (3/8)		37.5% (3/8)		25.0% (2/8)		12.5% (1/8)	
Pathology	Grade 3/4	33.3% (14/42)	0.543	28.6% (12/42)	0.478	21.4% (9/42)	0.100	16.7% (7/42)	0.422
	Grade 1/2	27.0% (10/37)		21.6% (8/37)		8.1% (3/37)		8.1% (3/37)	

*Statistically significant.

^#^Fisher’s exact test.

**Minimal subcortical monopolar threshold.

GTR, gross-total resection.

MEP, motor evoked potential.

SEP, somatosensory evoked potential

### Non-Impact of Minimal Subcortical Monopolar Threshold on Removal Degree of Gliomas

**Figure 2:**
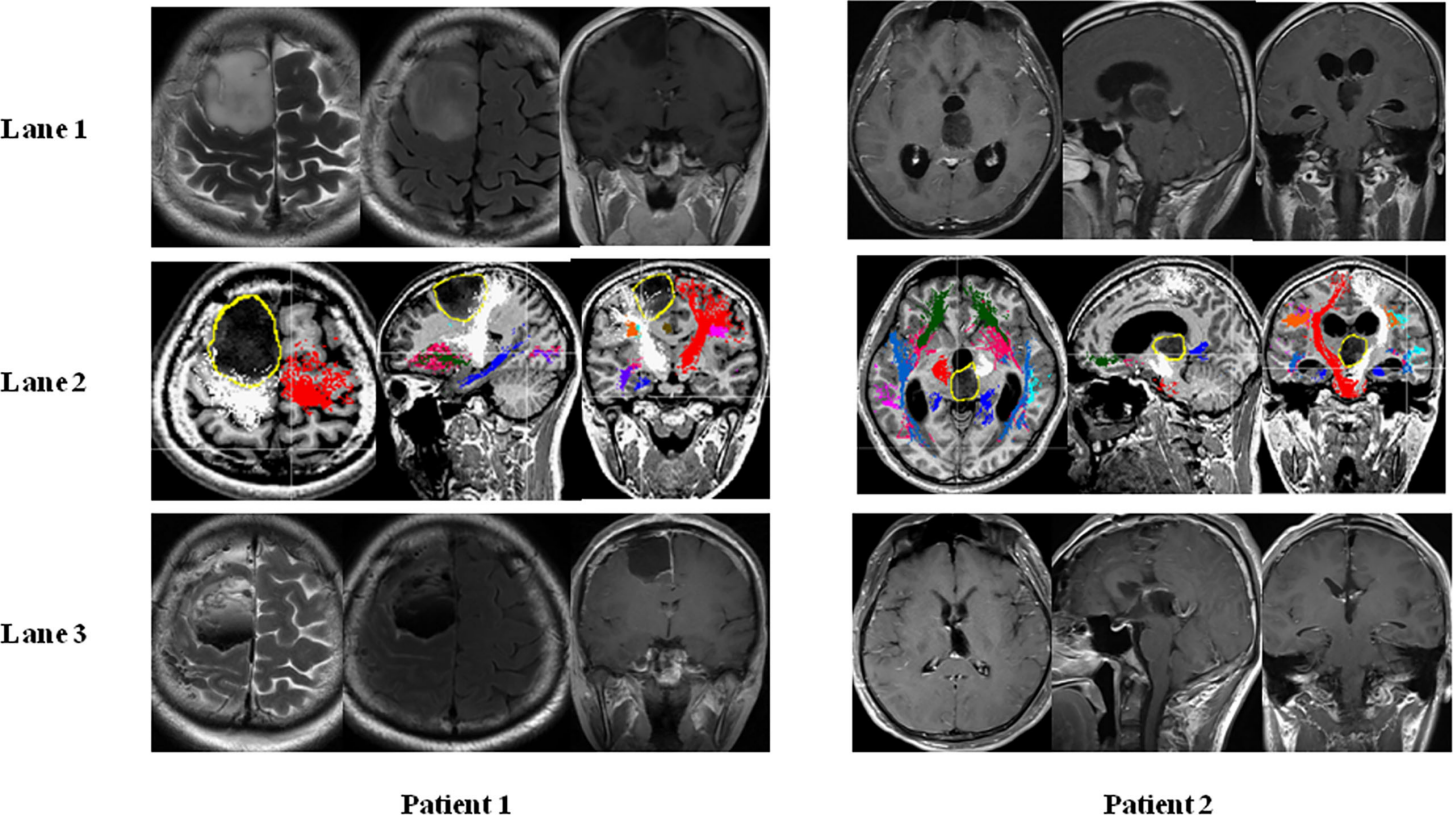
Minimal subcortical monopolar threshold (MSCMT) and the proximity to the motor pathway in patient 1 (astrocytoma in the supplement area with MSCMT = 3.4 mA and motor deficit after surgery) and patient 2 (anaplastic astrocytoma in the thalamus with MSCMT = 5.6 mA and no motor deficit after surgery). MR images are shown in lane 1 (before surgery), lane 2 [the proximity of tumor with the pyramid tract in the diffusion tensor imaging (DTI)], and lane 3 (after surgery).

To clarify the impact of MSCMT on the removal degree, we compared the MSCMT between GTR and non-GTR groups [5.6 mA (2.6–8.5) vs. 4.0 mA (2.2–8.3), *P* = 0.433]. The percentage of GTR was 62.5% (30/48) in the MSCMT >3.9 mA group and 54.8% (17/31) in the MSCMT ≤3.9 mA group (*P* = 0.498). The percentage of GTR was 53.7% (22/41) in the MSCMT ≤5.2 mA group and 65.8% (25/38) in the MSCMT >5.2 mA group (*P* = 0.272). The same results were also found in the low- and high-grade gliomas, respectively (data were not shown) (**Figure 2**).

### Obvious Relation of Minimal Subcortical Monopolar Threshold With Shorter Survivals in High-Grade Gliomas

**Figure 3:**
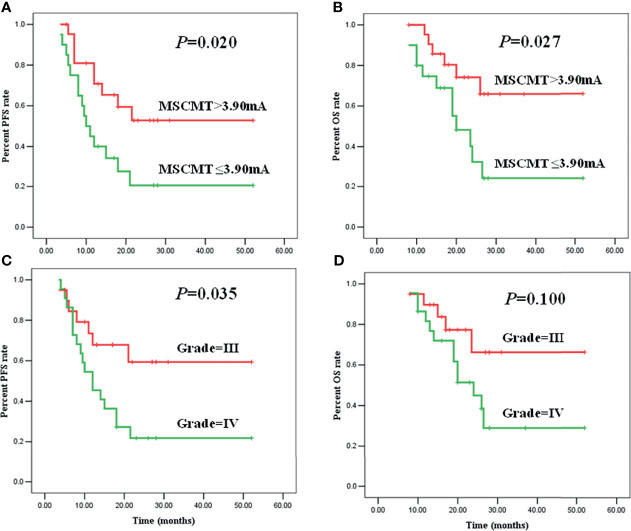
Minimal subcortical monopolar threshold (MSCMT) predicted shorter progression-free survival (PFS) [**(A)**, *P* = 0.020] and overall survival (OS) [**(B)**, *P* = 0.027]. Higher tumor grade (IV/III) predicted shorter PFS [**(C)**, *P* = 0.035] and OS [**(D)**, *P* = 0.100].

The median PFS and OS were 18.0 (95% CI 10.0–26.0) and 26.0 (95% CI 21.9–30.1) months for high-grade glioma patients (WHO grades III and IV). In high-grade gliomas, the association between clinical factors and survivals was analyzed by log-rank method and Cox regression models (**Tables 2**, **3** **;** **Figure 3**). Univariate Cox analysis revealed that WHO grades IV/III, IDH wild-type/mutant-type, and MSCMT ≤3.9 mA/>3.9 mA were correlated with PFS and OS. Multivariable Cox regression model confirmed that both WHO grades IV/III [hazard ratio (HR) = 3.296 (1.320–8.230), *P* = 0.011] and MSCMT ≤3.9 mA/>3.9 mA [HR = 3.381 (1.416–8.076), *P* = 0.006) were independently related to shorter PFS. Multivariable Cox regression model confirmed that both WHO grades IV/III [HR = 3.095 (1.077–8.894), *P* = 0.036) and MSCMT ≤3.9 mA/>3.9 mA [HR = 3.651 (1.336–9.977), *P* = 0.012] were independently related with shorter OS.

**Table 2:** Factors correlated with PFS and OS in high-grade gliomas (n = 42).

Factors	Progression-free survival (months)			Overall survival (months)		
	OR	95% CI	*P* value	OR	95% CI	*P* value
Age ≥40/<40 years	1.716	0.732–4.025	0.214	1.436	0.539–3.830	0.469
Women/Men	0.808	0.361–1.809	0.605	0.758	0.299–1.925	0.561
Primary/recurrent	1.950	0.458–8.300	0.366	2.356	0.313–17.739	0.405
Pre-op. strength weakness/normal	1.845	0.750–4.540	0.183	1.940	0.724–5.198	0.188
Motor deficit on day 1 after surgery	1.270	0.554–2.912	0.572	1.476	0.570–3.823	0.423
Motor deficit on day 7 after surgery	1.676	0.730–3.851	0.224	1.964	0.755–5.106	0.166
MSCMT ≤3.90 mA/>3.9 mA*	2.557	1.111–5.882	0.027	2.856	1.068–7.635	0.036
MSCMT ≤5.20 mA/>5.2 mA	2.190	0.952–5.039	0.065	2.528	0.908–7.035	0.076
Transcranial SEP decreased >50%	2.388	0.723–7.890	0.153	2.486	0.724–8.534	0.148
Tumor size ≥5 cm/<5 cm	1.138	0.509–2.545	0.753	1.436	0.555–3.716	0.455
Non-GTR/GTR	0.636	0.276–1.465	0.288	0.569	0.219–1.482	0.248
WHO grade IV/grade III	2.475	1.024–5.982	0.044	2.311	0.821–6.507	0.113
MGMT promoter methylation	0.794	0.348–1.815	0.585	0.843	0.317–2.240	0.731
IDH 1/2 mutation/wild-type	0.175	0.041–0.751	0.019	0.253	0.058–1.112	0.069

Transcranial MEP was excluded for limited cases with decreased MEP (n = 2).

*Minimal subcortical monopolar threshold.

PFS, progression-free survival.

OS, overall survival.

GTR, gross-total resection.

MGMT, O-6-methylguanine DNA methyltransferase.

IDH, isocitrate dehydrogenase.

**Table 3:** Independent factors to predict postoperative motor deficit (Logistic regression, n = 79) and prognoses (Cox regression, n = 42).

Independent factors to predict	OR (95% CI)	*P* value	Independent factors to predict	OR (95% CI)	*P* value
**Motor deficit on day 1 after surgery**			**Motor deficit on day 1 after surgery**		
Pre-op. strength weakness	7.217 (1.615–32.241)	0.010	Pre-op. strength weakness	8.635 (1.939–38.446)	0.005
MSCMT ≤3.9 mA/>3.9 mA	3.478 (1.192–10.145)	0.022	MSCMT ≤5.2 mA/>5.2 mA	3.125 (1.034–9.445)	0.043
**Motor deficit on day 7 after surgery**			**Motor deficit on day 7 after surgery**		
MSCMT ≤3.9 mA/>3.9 mA	4.230 (1.447–12.370)	0.008	Pre-op. strength weakness	4.418 (1.104–17.690)	0.036
			MSCMT ≤5.2 mA/>5.2 mA	3.688 (1.145–11.880)	0.029
**Motor deficit 3 months after surgery**			**Motor deficit 3 months after surgery**		
MSCMT ≤3.9 mA/>3.9 mA	3.826 (1.041–14.065)	0.043	MSCMT ≤5.2 mA/>5.2 mA	5.806 (1.181–28.540)	0.030
**Motor deficit 6 months after surgery**			**Motor deficit 6 months after surgery**		
MSCMT ≤3.9 mA/>3.9 mA	4.375 (1.036–18.473)	0.045	MSCMT ≤5.2 mA/>5.2 mA	10.406 (1.250–86.650)	0.030
**Shorter PFS**					
MSCMT ≤3.9 mA/>3.9 mA	3.381 (1.416–8.076)	0.006			
WHO grade IV/III	3.296 (1.320–8.230)	0.011			
**Shorter OS**					
MSCMT ≤3.9 mA/>3.9 mA	3.651 (1.336–9.977)	0.012			
WHO grade IV/III	3.095 (1.077–8.894)	0.036			

MSCMT, minimal subcortical monopolar threshold.

PFS, progression-free survival.

OS, overall survival.

### Quantitative Relationship Between Paired Monopolar and Bipolar Thresholds

To clarify the quantitative relationship between paired monopolar and bipolar thresholds, different mathematical models were calculated. Power model has the best fitness of all the tested models (see **Supplementary Table S3**, adjusted R square = 0.754, *P* < 0.001). The power (Y = 1.583X^0.944^) and linear (Y = 1.205X+2.334) models were plotted in **Supplementary Figure S1A** with Y=X as the reference line. When the monopolar stimulating current was no more than 10.0 mA, most points converged around the reference line, which meant that monopolar and bipolar threshold values were similar. However, when the monopolar stimulating current was more than 10.0 mA, most points deviated above from the reference line, which meant that bipolar threshold value was higher than monopolar threshold value in the same tissue location.

Monopolar stimulus is more sensitive than bipolar stimulus. In 13 of 89 threshold pairs, the bipolar provoking potentials were not detected under 40 mA. For the other pairs (N = 76), the mean monopolar provoking potential was significantly lower than the mean bipolar provoking potentials (7.7 ± 6.70 mA vs. 11.6 ± 9.92mA, paired t-test, *P* < 0.001, **Supplementary Figure S1B**).

## Discussion

For direct electrical stimulation in search of the motor pathway, two techniques have been developed: the low-frequency (50 or 60 Hz) bipolar stimulation first described by Penfield in 1937 (12) and the high-frequency multipulse train stimulation technique first described by Taniguchi in 1993 (13). High-frequency monopolar stimulation and low-frequency bipolar stimulation are two promising methods (14). The former preferentially activates the axons of the fast-conducting myelinated fibers of the corticospinal tract (CST) (15, 16) and is more effective than bipolar in subcortical activation of the CST (14). The subcortical threshold means the minimal distance away from the motor pathway and is a critical parameter to delineate the functional brain–tumor interface. In this study, monopolar thresholds were prospectively recorded and motor outcome was carefully followed in 79 patients. We discovered the relationship of MSCMT cutoff value with motor deficit and further analyzed the relationship between MSCMT and clinical prognoses.

### The Minimal Subcortical Monopolar Threshold Cutoff Value Related to Postoperative Motor Deficit

Subcortical stimulation has been used to map the CST, and the stimulation threshold may predict postoperative motor deficits. However, the study exploring the optimal MSCMT value to guide operation procedure without functional deficits was still limited. The referential conditions of interrupting resection ranged from the presence of a positive motor response regardless of applied stimulation frequency to stimulation thresholds between 5 and 7 mA (17–20). Our data revealed that the cutoff threshold to diagnose motor deficit in advance at different time points after surgery was 3.9 mA (on day 1), 3.7 mA (on day 7), 5.2 mA (3 months), and 5.2 mA (6 months). So, the safety threshold ranged from 3.9 to 5.2 mA. Here, 3.9mA is the safe cutoff MSCMT to predict motor deficit during operation procedure. Our data revealed that MSCMT ≤3.9 mA independently predicted motor deficit at different time points after surgery. Near the CST, MSCMT >3.9 mA predicted a favorable motor outcome. For patients with MSCMT beyond the safety range, we suggested hyperbaric oxygen and rehabilitation training as soon as possible.

Keles et al. (18) reported that 7.3% of patients with detected subcortical pathways experienced permanent motor deficits compared with only 2.1% of those with undetected subcortical pathways, with a current varying from 1 to 16 mA. Nossek et al. (17) found that 5 of 7 patients with late postoperative deterioration (>5 days postoperatively) or no recovery had subcortical high-frequency monopolar stimulus threshold <3 mA (sensitivity of 83% and specificity of 95%). Seidel et al. (21) found that irreversible direct cortical MEP changes and motor deficits regularly occurred with a subcortical monopolar threshold of 1 mA or less and recommended aborting tumor resection at a threshold of 2 mA. Mikuni et al. (19) found that a distance <7 mm correlated with positive motor responses, while distances beyond 13 mm did not produce a motor response with 50-Hz bipolar stimulus at a constant intensity. Prabhu et al. (22) found a trend toward worsening neurological deficits if the distance from the probe to the CST was short (<5 mm), indicating close proximity of the resection cavity to the CST.

MSCMT is the golden criteria for the mapping of CST and is superior to the radiological mapping of CST, especially during the operation procedures. The radiological mapping of CST including the functional MR and DTI is useful to locate the CST. However, the accurate identification of CST by radiological image is less objective and less exact than the MSCMT, especially due to the navigation error by brain shift.

### Less Minimal Subcortical Monopolar Threshold Implicated the Shorter Survivals in High-Grade Gliomas

With the long-term following up, we reported that MSCMT ≤3.9 mA was associated with shorter survivals in high-grade gliomas. MSCMT mostly reflected the distance away from CST. Lower MSCMT implied the more proximity or infiltration to the motor pathway, as a prognostic characteristic of the malignant high-grade gliomas. Lower MSCMT may also result in more motor deficits or non-GTR, which also attributed to the worse prognoses. Besides, we compared the MSCMT between GTR and non-GTR groups and found no significant difference. We found no correlation between removal degree and prognoses either. It is reported that gliomas with the subventricular or the putamen involvement is with worse prognosis than those without the involvement (23–25). Most gliomas with lower MSCMT have such involvement, which might account for the worse prognoses. We will make further analysis to explain why smaller MSCMT predicts shorter survivals.

### Comparison of Paired Monopolar and Bipolar Thresholds on the Subcortex

To clarify the quantitative relationship between paired monopolar and bipolar threshold values, we established the power regression model for paired monopolar and bipolar threshold values, which represented better fitness than the linear model. According to this positively correlated model, we could calculate the bipolar threshold from the monopolar threshold and *vice versa*.

Our data revealed that monopolar stimulation is more sensitive than bipolar stimulation for CST mapping. Szelenyi et al. (14) revealed that monopolar multipulse stimulation had the lowest stimulation intensities for MEPs and elicited MEPs in a higher number of tested patients than bipolar stimulation. Nossek et al. (17) concluded that subcortical stimulation with a monopolar probe and high-frequency multi-pulse stimulation is the most efficient to identify the CST. Gogos et al. (26) reported that subcortical motor pathways were identified in 51 cases (86.4%) with monopolar high-frequency stimulation, but only in 6 patients using bipolar stimulation. Gomez-Tames et al. (27) compared monopolar and bipolar stimulation and found that bipolar stimulation could produce more selective activation if the CST was close to the resection border and monopolar stimulation was more robust and more effective for the CST far from the stimulation point. The different current spreading modes may account for the different sensitivities. Previous studies in primates revealed that the current spread associated with the bipolar stimulation modality is limited to 2 or 3 mm (28). Therefore, the MEP may not be easily elicited with bipolar stimulus at the point far from the CST, which explained that the bipolar stimulus was less sensitive than the monopolar stimulus.

### Study Limitations

Firstly, biases exist due to the small sample size in the study, and a larger cohort will be needed to further test these findings. Secondly, we make a presumption that all the patients are similar in the aspect of tumor pathology and texture. A further study enrolling more patients with gliomas involving CST is needed to validate our findings.

### Conclusions

The MSCMT, as a promising reference to guide operation procedure of gliomas involving CST among non-awake patients, has not been reported discreetly. In this study, we explored the cutoff value of MSCMT and analyzed its potential diagnostic value in predicting postoperative motor deficit and long-term survivals. The MSCMT cutoff value related to motor deficit at different time points, ranging from 3.7 to 5.2 mA. Lower MSCMT independently predicted postoperative motor deficits and shorter survivals.

## Data Availability Statement

The original contributions presented in the study are included in the article/**Supplementary Material**. Further inquiries can be directed to the corresponding authors.

## Ethics Statement

The studies involving human participants were reviewed and approved by Medical Ethics Committee of Beijing Tiantan Hospital. The patients/participants provided their written informed consent to participate in this study.

## Author Contributions

Experimental design: XR, SL, and LG. Implementation: XR, XY, WH, LL, KY, YC, HQ, LG, and SL. Analysis and interpretation of the data: XR, XY, WH, LL, KY, LG, and SL. Article writing at draft and revision, approved the final version: all authors.

## Funding

This work was supported by Capital Health Research and Development of Special of Beijing (2014-2-2042) and the National Natural Science Foundation of China (81401381&81771309).

## Conflict of Interest

The authors declare that the research was conducted in the absence of any commercial or financial relationships that could be construed as a potential conflict of interest.

## Publisher’s Note

All claims expressed in this article are solely those of the authors and do not necessarily represent those of their affiliated organizations, or those of the publisher, the editors and the reviewers. Any product that may be evaluated in this article, or claim that may be made by its manufacturer, is not guaranteed or endorsed by the publisher.

## Supplementary Material

Supplementary Figure 1
**(A)** The power and linear models for paired sub-cortical monopolar and bipolar threshold values at the same point with Y=X as a reference. **(B)** The mean monopolar threshold was lower than the paired bipolar threshold for sub-cortical stimulation (paired t-test, *P *< 0.001).

## Abbreviations

MR, magnetic resonance; CST, cortico-spinal tract; MSCMT, minimal sub-cortical monopolar threshold; PFS, progression-free survival; OS, overall survival.
