# Supplementary figures and images for: The Minimal Subcortical Electronic Threshold Predicts the Motor Deficit and Survivals in Non-Awake Surgery for Gliomas Involving the Motor Pathway
Source: Front Oncol. 2022 Mar 15;12:789705. doi: 10.3389/fonc.2022.789705 (PMC8965070; doi:10.3389/fonc.2022.789705)

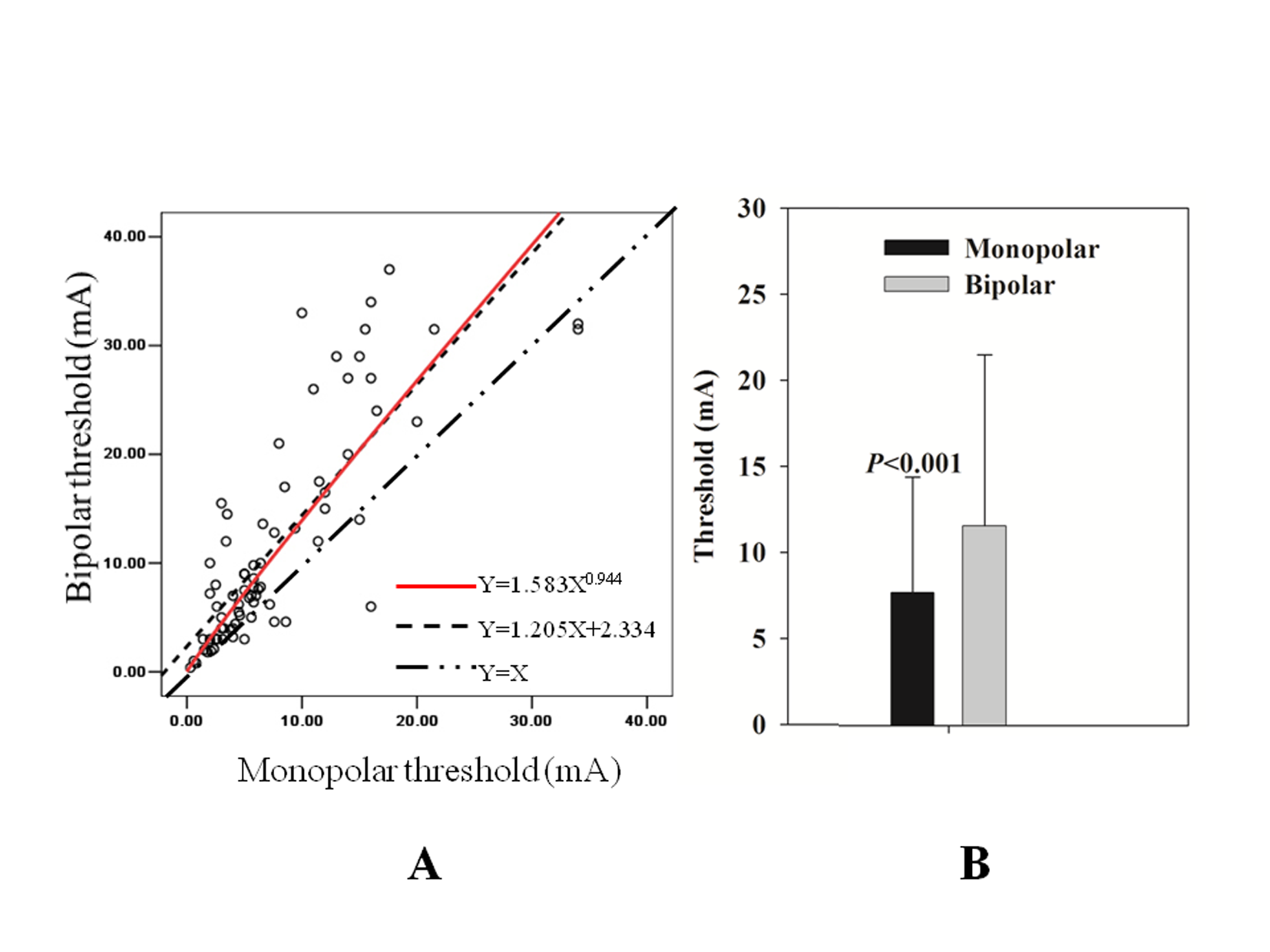

Supplement: Supplementary Figure 1 — (A) The power and linear models for paired sub-cortical monopolar and bipolar threshold values at the same point with Y=X as a reference. (B) The mean monopolar threshold was lower than the paired bipolar threshold for sub-cortical stimulation (paired t-test, P < 0.001). [file Image_1.tif]
