# Supplementary material for: The Minimal Subcortical Electronic Threshold Predicts the Motor Deficit and Survivals in Non-Awake Surgery for Gliomas Involving the Motor Pathway
Source: Front Oncol. 2022 Mar 15;12:789705. doi: 10.3389/fonc.2022.789705 (PMC8965070; doi:10.3389/fonc.2022.789705)
Supplement: Supplementary file 2 [file Table_1.docx]

**TABLE S1: Clinical characteristics of 79 patients with motor pathway gliomas**

| **Characteristic** |  | **Value (%)*** |
| --- | --- | --- |
| Age at diagnosis in yrs | Mean | 42± 14 |
|  | Range | 14-75 |
| Gender | M | 44 (55.7) |
|  | F | 35 (44.3) |
| Preoperative KPS | Median | 80 |
|  | Range | 40-100 |
| Primary or recurrent | Primary | 71 (89.9) |
|  | Recurrent | 8 (10.1) |
| Tumor location | Insular | 26 (32.9) |
|  | Frontal/temporal/parietal (perirolandic) | 46 (58.2) |
|  | Thalamus | 7 (8.9) |
| Tumor sides | Left | 43 (54.4) |
|  | Right | 36 (45.6) |
| Tumor size (cm) | Mean | 4.6±1.7 |
|  | Range | 1.0-9.0 |
| Pathological diagnosis | Grade I | 3 (3.8) |
|  | Grade II | 34 (43.0) |
|  | Grade III | 20 (25.3) |
|  | Grade IV | 22 (27.8) |
| IDH1/2 mutation | Yes | 43 (54.4) |
|  | No | 36 (45.6) |
| MGMT promoter methylation | Yes | 52 (65.8) |
|  | No | 19 (24.1) |
|  | N/A | 8 (10.1) |
| Removal degree | GTR | 47 (59.5) |
|  | STR | 28 (35.4) |
|  | Partial | 4 (5.1) |
| Pre-op. strength of limb | Normal | 68 (86.1) |
|  | Weakened | 11 (13.9) |
| Motor function (1^st^ day post-op.) | Improved/Unchanged | 55 (69.6) |
|  | Worsened | 24 (30.4) |
| Motor function (7^st^ day post-op.) | Improved/Unchanged | 59 (74.7) |
|  | Worsened | 20 (25.3) |
| Motor function (3 months post-op.) | Improved/Unchanged | 67 (84.8) |
|  | Worsened | 12 (15.2) |
| Motor function (6 months post-op.) | Improved/Unchanged | 69 (87.3) |
|  | Worsened | 10 (12.7) |
| MSCMT** (mA) | Range | 0.1-27.0 |
|  | Median | 5.0 |
| Transcranial MEP | Unchanged | 47 (59.5) |
|  | Decreased | 2 (2.5) |
|  | Unavailable | 30 (38.0) |
| Transcranial SEP | Unchanged | 43 (54.4) |
|  | Decreased | 8 (10.1) |
|  | Unavailable | 28 (35.4) |

*All means are expressed ± SD, ** Minimal sub-cortical monopolar threshold
