# Supplementary material for: The Minimal Subcortical Electronic Threshold Predicts the Motor Deficit and Survivals in Non-Awake Surgery for Gliomas Involving the Motor Pathway
Source: Front Oncol. 2022 Mar 15;12:789705. doi: 10.3389/fonc.2022.789705 (PMC8965070; doi:10.3389/fonc.2022.789705)
Supplement: Supplementary file 3 [file Table_2.docx]

**Table S2. The cutoff monopolar threshold to predict motor deficit at different time points after surgery (N=79)**

| **After surgery** | **Day 1** | **Day 7** | **3 months** | **6 months** |
| --- | --- | --- | --- | --- |
| Cutoff threshold | 3.90mA | 3.7mA | 5.2mA | 5.2mA |
| Maximal AUC (95% CI) | 0.672 (0.541-0.804) | 0.716 (0.595-0.837) | 0.681 (0.536-0.826) | 0.708 (0.573-0.843) |
| P value | 0.015 | 0.004 | 0.047 | 0.034 |
| Sensitivity | 62.5% | 65.0% | 83.3% | 90% |
| Specificity | 70.9% | 71.2% | 53.7% | 53.6% |
| Positive predictive value | 48.4% | 43.3% | 24.4% | 22.0% |
| Negative predictive value | 81.3% | 85.7% | 94.7% | 97.4% |
