# Supplementary material for: The Minimal Subcortical Electronic Threshold Predicts the Motor Deficit and Survivals in Non-Awake Surgery for Gliomas Involving the Motor Pathway
Source: Front Oncol. 2022 Mar 15;12:789705. doi: 10.3389/fonc.2022.789705 (PMC8965070; doi:10.3389/fonc.2022.789705)
Supplement: Supplementary file 4 [file Table_3.docx]

**Table S3. Curve estimation of regression models for 76 paired mono- and bi-polar thresholds**

| Model types | R square | Adjusted R square | Unstandardized coefficients | | P value |
| --- | --- | --- | --- | --- | --- |
|  |  |  | B | Std. Error |  |
| Linear | 0.662 | 0.658 | 1.205 | 0.100 | <0.001 |
| Logarithmic | 0.600 | 0.594 | 8.628 | 0.820 | <0.001 |
| Inverse | 0.171 | 0.160 | -9.411 | 2.410 | <0.001 |
| Quadratic | 0.722 | 0.714 | 2.111 | 0.247 | <0.001 |
| Cubic | 0.730 | 0.719 | 1.154 | 0.678 | 0.093 |
| Compound | 0.537 | 0.530 | 1.111 | 0.013 | <0.001 |
| Power | 0.758 | 0.754 | 0.944 | 0.062 | <0.001 |
| S | 0.479 | 0.472 | -1.533 | 0.186 | <0.001 |
| Growth | 0.537 | 0.530 | 0.106 | 0.011 | <0.001 |
| Exponential | 0.537 | 0.530 | 0.106 | 0.011 | <0.001 |
| Logistic | 0.537 | 0.530 | 0.900 | 0.010 | <0.001 |
